# Predictive value of synaptic plasticity for functional decline in patients with multiple sclerosis

**DOI:** 10.3389/fneur.2024.1410673

**Published:** 2024-06-21

**Authors:** Carolin Balloff, Lisa Kathleen Janßen, Christian Johannes Hartmann, Sven Günther Meuth, Alfons Schnitzler, Iris-Katharina Penner, Philipp Albrecht

**Affiliations:** ^1^Department of Neurology, Medical Faculty and University Hospital Düsseldorf, Heinrich Heine University, Düsseldorf, Germany; ^2^Department of Neurology, Kliniken Maria Hilf GmbH, Mönchengladbach, Germany; ^3^Institute of Clinical Neuroscience and Medical Psychology, Medical Faculty and University Hospital Düsseldorf, Heinrich Heine University, Düsseldorf, Germany; ^4^Department of Neurology, Inselspital, Bern University Hospital, University of Bern, Bern, Switzerland

**Keywords:** synaptic plasticity, multiple sclerosis, repetitive transcranial magnetic stimulation, quadripulse stimulation, functional decline, disease progression

## Abstract

**Background:**

Previous research suggested that quadripulse (QPS)-induced synaptic plasticity is associated with both cognitive and motor function in patients with multiple sclerosis (MS) and does not appear to be reduced compared to healthy controls (HCs).

**Objective:**

This study aimed to explore the relationship between the degree of QPS-induced plasticity and clinically significant decline in motor and cognitive functions over time. We hypothesized that MS patients experiencing functional decline would exhibit lower levels of baseline plasticity compared to those without decline.

**Methods:**

QPS-induced plasticity was evaluated in 80 MS patients (56 with relapsing-remitting MS and 24 with progressive MS), and 69 age-, sex-, and education-matched HCs. Cognitive and motor functions, as well as overall disability status were evaluated annually over a median follow-up period of 2 years. Clinically meaningful change thresholds were predefined for each outcome measure. Linear mixed-effects models, Cox proportional hazard models, logistic regression, and receiver-operating characteristic analysis were applied to analyse the relationship between baseline plasticity and clinical progression in the symbol digit modalities test, brief visuospatial memory test revised (BVMT-R), nine-hole peg test (NHPT), timed 25-foot walk test, and expanded disability status scale.

**Results:**

Overall, the patient cohort showed no clinically relevant change in any functional outcome over time. Variability in performance was observed across time points in both patients and HCs. MS patients who experienced clinically relevant decline in manual dexterity and/or visuospatial learning and memory had significantly lower levels of synaptic plasticity at baseline compared to those without such decline (NHPT: *β* = −0.25, *p* = 0.02; BVMT-R: *β* = −0.50, *p* = 0.005). Receiver-operating characteristic analysis underscored the predictive utility of baseline synaptic plasticity in discerning between patients experiencing functional decline and those maintaining stability only for visuospatial learning and memory (area under the curve = 0.85).

**Conclusion:**

Our study suggests that QPS-induced plasticity could be linked to clinically relevant functional decline in patients with MS. However, to solidify these findings, longer follow-up periods are warranted, especially in cohorts with higher prevalences of functional decline. Additionally, the variability in cognitive performance in both patients with MS and HCs underscores the importance of conducting further research on reliable change based on neuropsychological tests.

## 1 Introduction

Multiple sclerosis (MS) is a neurological disease, characterized by inflammatory, demyelinating lesions and neurodegeneration (1), leading to a wide range of motor, sensory, and cognitive impairments (2). For most patients (~85%), the disease manifests as relapsing-remitting MS (RRMS), which is characterized by sudden episodes of new or exacerbated neurological symptoms alternating with periods of symptom remission and clinical stability (3). Over time, frequency of symptom remission decreases, and a majority of untreated RRMS patients (>80%) progress to secondary progressive MS (SPMS) within 25 years (4). SPMS involves a progressive worsening of symptoms with or without acute exacerbations (5). In contrast to RRMS and SPMS, individuals with primary progressive MS (PPMS) do not experience acute exacerbations. Instead, symptoms increase gradually starting from disease onset (6). PPMS affects approximately 10–20% of MS patients (7).

The mechanisms contributing to disability accumulation in MS are still not fully understood (8) and are discussed controversially. Despite efforts to identify biomarkers indicative of disease activity and progression, their clinical utility at the individual level is constrained (9). This challenge has been called attention to by the long-standing recognition of the “clinico-radiological paradox,” which underscores the discrepancies between observed lesion burden in brain imaging and clinical symptom presentation (10). In addition to brain and cognitive reserve (11), this paradox might be attributed to neuroplasticity, i.e., the brain’s ability to adapt and reorganize (12, 13). Neuroplasticity may compensate for structural damage, albeit to a diminishing extent as the disease advances. Although neuroplasticity involves several aspects, reorganization at the synaptic level through the reinforcement or weakening of synapses, known as long-term potentiation (LTP) or long-term depression (LTD), respectively, is a key component of synaptic plasticity (14).

One emerging avenue of investigating synaptic plasticity non-invasively involves the application of quadripulse stimulation (QPS), a transcranial magnetic stimulation (TMS) protocol known for its ability to induce both LTP and LTD in healthy subjects (15). We have previously investigated the functional relevance of LTP-like plasticity induced by QPS in patients with MS cross-sectionally and revealed that levels of QPS-induced plasticity correlate with cognitive and motor function among individuals with intact pyramidal tract integrity (16). Importantly, the level of LTP-like plasticity was not reduced in patients of all disease types compared to healthy controls (HCs) (16, 17).

Previous studies regarding the clinical relevance of LTP-like plasticity for MS disease progression have relied solely on cross-sectional designs and revealed conflicting results. One comparison of LTP-like plasticity between RRMS and PPMS patients suggested a potential association between diminished levels of plasticity and disease progression (18). In contrast, our attempt to replicate this finding using QPS did not confirm it, but instead indicated comparable levels of LTP-like plasticity across both groups (16). Furthermore, another study found that enhanced synaptic plasticity after 4 weeks of oral D-aspartate treatment was not associated with improvements in clinical outcomes (19). To date, longitudinal studies regarding the clinical relevance of LTP-like plasticity for MS disease progression are lacking to the best of our knowledge. However, research on the transition to dementia in individuals with memory impairment has proposed that LTP-like plasticity could potentially serve as a predictive biomarker for clinical progression (20).

In summary, cross-sectional studies have yielded inconclusive results regarding the clinical relevance of LTP-like plasticity for MS disease progression. Longitudinal studies are warranted to comprehensively explore this aspect. Therefore, the objective of this study was to examine the relationship between the degree of LTP-like plasticity at baseline and disease progression up to 5 years after QPS assessment in patients with MS. Drawing from previous research that has suggested a potential association between QPS-induced plasticity and clinical outcomes (16, 17), and considering the promising results in the field of dementia (20), we hypothesized that patients with lower baseline plasticity levels would exhibit greater disease progression compared to those with higher plasticity levels.

## 2 Materials and methods

### 2.1 Subjects

Patients diagnosed with definite MS according to the 2017 revised McDonald criteria (21) were enrolled in the study, along with age-, sex-, and education-matched HCs. Monocentric recruitment of patients occurred at the neurological clinic of the University Hospital Düsseldorf, Germany, from May 2018 to October 2022. HCs were recruited as a convenience sample from an internal database of interested HCs, friends, and family members of faculty members of the University Hospital Düsseldorf. All participants were invited for annual follow-ups for up to 5 years after baseline assessment.

Participants with at least one follow-up after baseline assessment were included. Exclusion criteria comprised a history of neurological or psychiatric disorders other than MS and remitted depressive episodes at baseline or follow-up. Additional exclusion criteria included contraindications for TMS and substance or alcohol abuse, which were assessed through a TMS safety screening questionnaire (22). At baseline and each follow-up, patients were required to be relapse free for ≥30 days and appointments were postponed in case patients did not fulfill this requirement.

The study received ethical approval from the ethical committee of the medical faculty of Heinrich Heine University Düsseldorf (study-number 2018-16), and informed written consent was obtained from all participants before study participation. The study adhered to the principles of the Declaration of Helsinki.

### 2.2 Experimental designs and procedure

**Figure 1:**
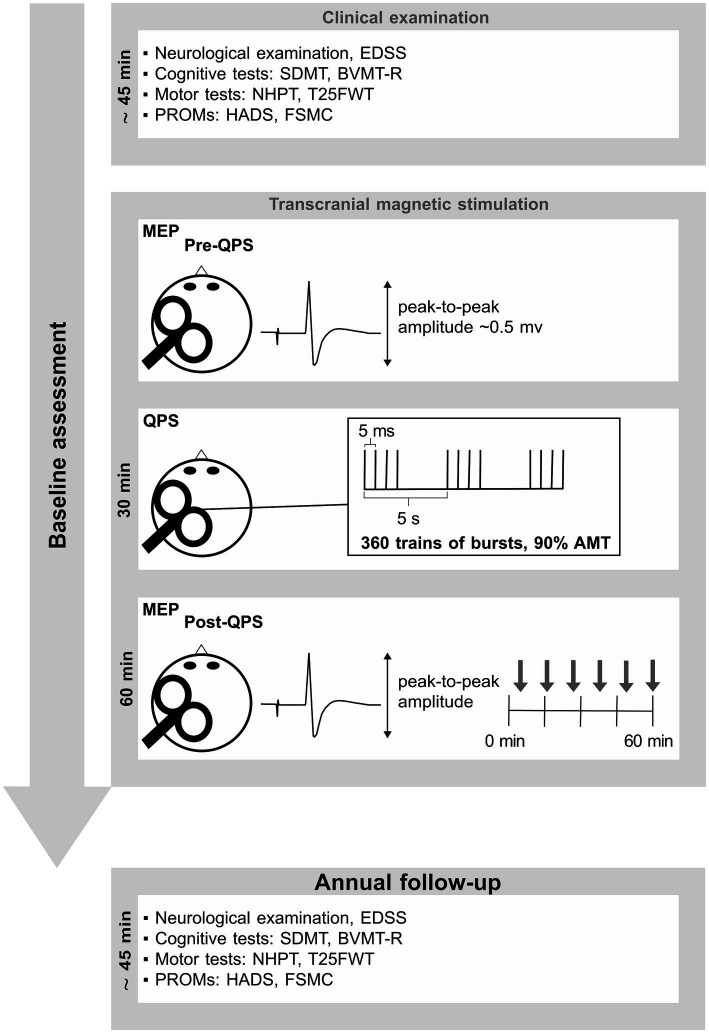
This figure summarizes the experimental design. Baseline assessment consisted of a short neuropsychological test battery, including PROMs. Details are described in our previous publications (16, 17, 23). EDSS, expanded disability status scale; SDMT, symbol digit modalities test; BVMT-R, brief visuospatial memory test-revised; NHPT, nine-hole peg test; T25FWT, timed 25-foot walk test; PROMs, patient reported outcome measures; QPS, quadripulse stimulation; AMT, active motor threshold; HADS, hospital anxiety and depression scale; FSMC, fatigue scale for motor and cognitive functions. MEP, motor evoked potential.

The experimental design of this longitudinal study is summarized in Figure 1. At baseline, data were assessed according to the procedures described in our previous studies (16, 17, 23). To summarize, baseline assessment was divided into five parts: (1) a neurological examination, (2) a neuropsychological examination, (3) an assessment of motor function, (4) patient reported outcome measures (PROMS), and (5) assessment of QPS-induced synaptic plasticity using a faciliatory protocol (interstimulus interval 5 ms, QPS-5) (24).

The neurological examination included an assessment of the expanded disability status scale (EDSS) to gauge the extent of disability attributed to MS. The neuropsychological assessment encompassed the symbol digit modalities test (SDMT) (25) as a measure of information processing speed, and the brief visuospatial memory test revised (BVMT-R) (26) as a measure of visuospatial short-term memory and learning. Motor function was assessed using the nine-hole peg test (NHPT) to evaluate manual dexterity and fine motor skills, while the timed 25-foot walk (T25FWT) was used as a measure of walking ability (27). PROMs comprised the hospital anxiety and depression scale (HADS) (28) as a measure of anxiety and depression, and the fatigue scale for motor and cognitive functions (FSMC) (29) as a measure of trait fatigue. QPS-induced plasticity was operationalized by the change in motor evoked potential (MEP) amplitudes recorded at the right first dorsal interosseous muscle following QPS-5 of the contralateral motor cortex. MEP amplitude prior to QPS-5 was adjusted to be ~0.5 mV in all participants. After 30 min of QPS-5, MEP responses evoked by the same pre-interventional stimulation intensity were recorded for a total of 60 min at the same muscle. At each time of assessment, it was intended to average 12 MEPs. However, due to artifacts or voluntary muscle activity, certain MEPs had to be excluded from the calculation of the averaged MEP amplitude, resulting in a median of 11 averaged MEPs for each time point and subject.

Our objective was to carry out in-person assessments during follow-ups. However, unforeseen circumstances (e.g., COVID-19 pandemic) occasionally resulted in participants being unavailable for in-person appointments. In such instances, remote follow-up assessments using a video conferencing tool were conducted, consisting of a structured interview, followed by cognitive tests. PROMs (HADS, FSMC) were filled out by the participant immediately after the video conference. No assessments of motor functions were conducted during remote follow-ups (see Supplementary material for details on remote assessment). Independent of assessment mode (remote vs. in-person), alternative forms of the BVMT-R were used at each follow-up to minimize practice effects.

### 2.3 Definition of clinically meaningful change

The objective of this study was to investigate the predictive value of synaptic plasticity for clinically meaningful change in cognition, motor function, and disability, as measured by EDSS. Since these outcomes are subject to day-to-day fluctuations, cut-off values to discriminate clinically meaningful change from expected measurement variability were required.

In line with common research practice, clinically meaningful change in the EDSS was defined as a change of ≥1 point for baseline scores ≤5.5 and a change of ≥0.5 point for baseline scores ≥6.0 (30, 31). For the SDMT, a change of ≥8 raw score points was considered clinically significant, since it has recently been demonstrated that this cut-off is more reliable than the previously used cut-off of ≥4 raw score points (32, 33). Regarding reliable change on the BVMT-R, we incorporated a cut-off of ≥8 points in the total learning trials, which has been presented at the 28th Congress of the European Committee for Treatment and Research in Multiple Sclerosis by the BICAMS initiative (34). For both the T25FWT and the NHPT, the well-established cut-off of a change of ≥20% was considered clinically significant (31, 35, 36).

### 2.4 Statistical analyses

Sample size was determined based on the number of eligible MS patients and matched HCs, since this is the first study investigating QPS-induced plasticity as a prognostic marker of disease progression in patients with MS. No imputation was performed to address missing data, except for participants who were unable to complete the T25FWT due to disability. In line with our previous study (16), imputation for patients unable to perform the T25FWT was based on the following formula:


$$\begin{array}{l} \mathrm{Time}\;\mathrm{in}\;\mathrm{ms}=\mathrm{maximum}\;\mathrm{time}\;\mathrm{within}\;\mathrm{the}\;\mathrm{total}\;MS\;\mathrm{cohort}\\ \mathrm{across}\;\mathrm{all}\;\mathrm{times}\;\mathrm{of}\;\mathrm{assessment}+1.645*SD\;\mathrm{within}\;\mathrm{the}\\ \mathrm{total}\;MS\;\mathrm{cohort}\;\mathrm{across}\;\mathrm{all}\;\mathrm{times}\;\mathrm{of}\;\mathrm{assessment} \end{array}$$

Chi-square test (SDMT, T25FWT) or Fisher’s exact test (NHPT, BVMT-R) was used to compare the number of subjects with clinically relevant decline/improvement between patients with MS and HCs. The absolute changes in each outcome from baseline to latest follow-up were compared among groups using Kruskal–Wallis test. Significant omnibus tests were followed by Dunn’s test to ascertain the specific group difference(s).

Linear mixed-effects models (LMEM) were employed for each functional outcome (SDMT, BVMT-R, NHPT, T25FWT, EDSS) to compare the level of baseline plasticity between patients with clinically relevant decline at latest FU and those without. Consistent with our prior cross-sectional investigations of QPS-induced plasticity in patients with MS (16, 17, 23) and given the good reproducibility and standardization of this approach (24), baseline synaptic plasticity was defined based on MEP amplitude changes from pre to post QPS (ΔMEP). The model consisted of fixed effects of the intervention (pre/post-QPS) and group classification (relevant decline/no relevant decline), as well as their interaction (QPS*group) and was estimated using the “restricted maximum likelihood method.” The subject-specific variability in response to QPS was considered by a random slope for the QPS intervention (pre/post-QPS). In the context of our hypothesis of reduced baseline plasticity in patients with clinically relevant decline at latest FU, the interaction (QPS*group) was of primary interest for each functional outcome, as significant interactions would suggest significant differences in the extent of plasticity among the respective groups. Significance of this effect was tested based on one-tailed confidence intervals and *p*-values of the QPS*group factor. Confidence intervals and *p*-values of all other factors were based on two-tailed analysis.

Consistent with our previous studies (16, 17, 23), additional factors such as age, depression, anxiety, fatigue, and MEP latency, along with their interactions with the QPS intervention, were separately introduced into the model. Furthermore, disease duration, EDSS at baseline, and time since baseline were introduced to account for different clinical baseline characteristics and different follow-up times. However, baseline EDSS was not introduced to the model comparing QPS-induced plasticity between patients with and without EDSS worsening, as baseline EDSS is a strong predictor of EDSS worsening with lower baseline EDSS being associated with more change over time (37, 38).

Each of these more complex models was compared against the simplest model through likelihood-ratio tests or, in instances of missing data in covariates, through the Akaike information criterion. Collinearity was assessed using the variance inflation factor (cutoff value of ≥5). The best fitting model is presented.

In addition to the LMEM, time to event analysis using Cox proportional hazard models correcting for age at baseline and sex, were conducted to compare the probability of clinically meaningful change in each functional outcome between patients with high and low baseline plasticity, which was defined based on a median split of ΔMEP. Event rates for both groups (high vs. low plasticity) were estimated using Kaplan–Meier analysis. Additionally, receiver-operating characteristic analysis and logistic regression were conducted to evaluate the ability of baseline plasticity to discriminate between patients with and without clinically relevant decline in the functional outcomes. Logistic regression was performed using plasticity at baseline (ΔMEP) and time since baseline as continuous predictors of functional decline (yes vs. no) in each outcome (SDMT, BVMT-R, NHPT, T25FWT, EDSS). To interpret the results of the receiver-operating characteristic analysis, the area under the curve was calculated.

The nlme package in R Studio (version 2023.12.1 + 402 for windows) were used to conduct LMEM. All other analyses were conducted using IBM SPSS Statistics (version 29.0.1.0 for windows).

## 3 Results

**Figure 2:**
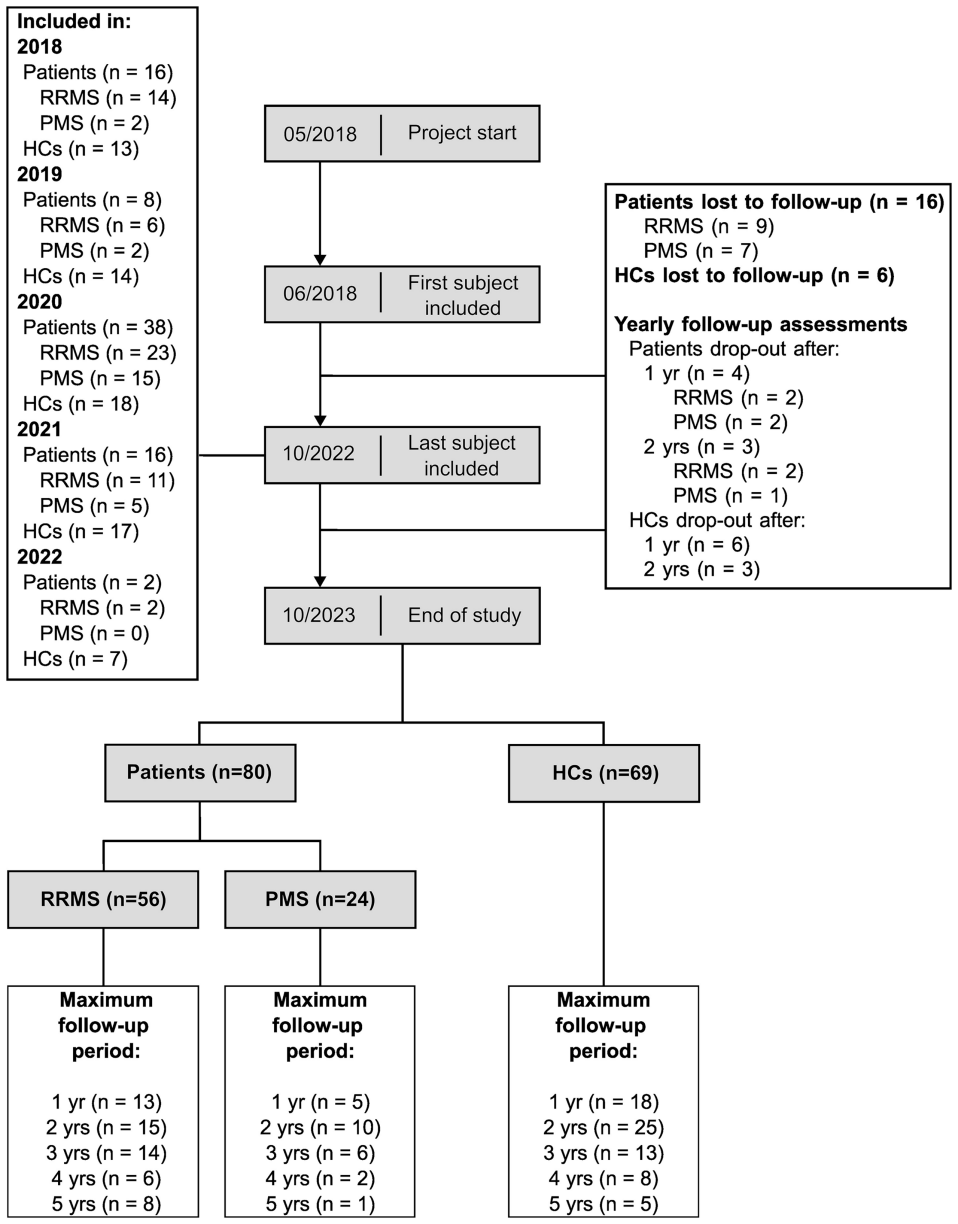
Longitudinal cohort flowchart. This figure summarizes the number of participants throughout the study. RRMS, patients with relapsing-remitting multiple sclerosis; PMS, patients with progressive multiple sclerosis; HCs, healthy controls; yr, year; yrs, years.

Out of 96 patients and 75 HCs included at baseline assessment, 56 patients with RRMS, 24 patients with PMS (14 SPMS, 10 PPMS) and 69 HCs completed at least one follow-up assessment (see Figure 2).

### 3.1 Neurological and neuropsychological trajectories

**Figure 3:**
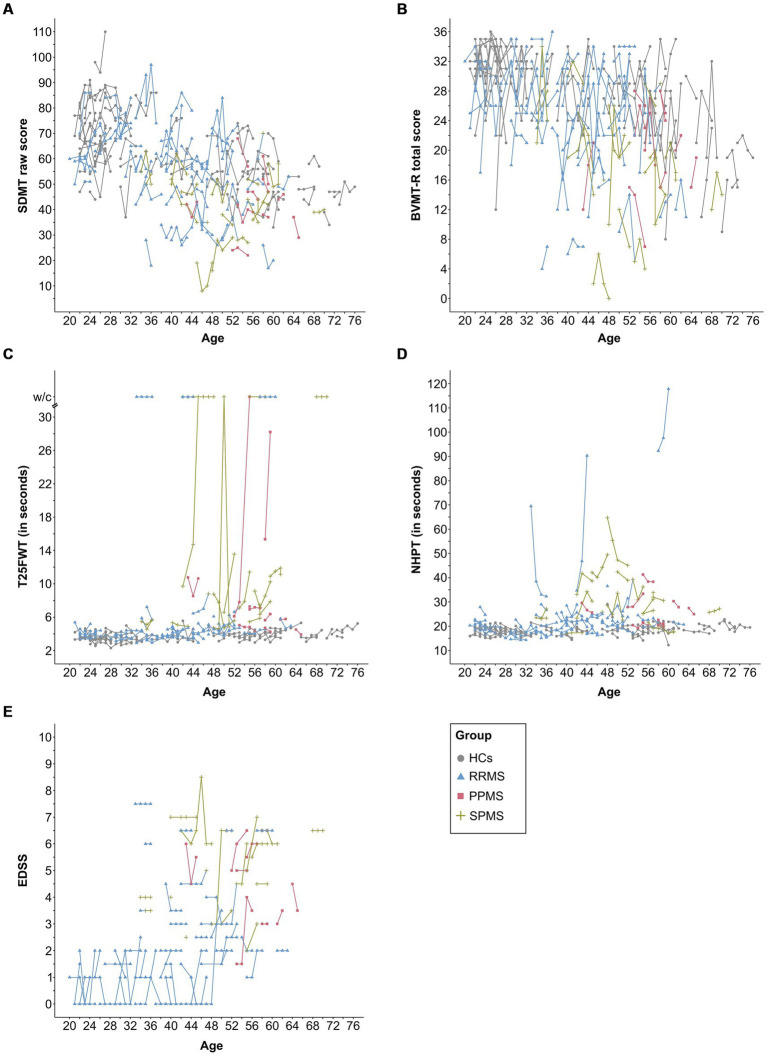
This figure displays the raw data for each assessment and time point per subject for the SDMT **(A)**, BVMT-R **(B)**, NHPT **(C)**, T25FWT **(D)**, and EDSS **(E)**. Lines connect data from the same subject. EDSS was missing for one patient (SPMS) at baseline. SDMT, symbol digit modalities test; BVMT-R, brief visuospatial memory test-revised; NHPT, nine-hole peg test; T25FWT, timed 25-foot walk test; w/c, wheelchair; EDSS, expanded disability status scale; PPMS, patients with primary progressive multiple sclerosis; SPMS, patients with secondary progressive multiple sclerosis; RRMS, patients with relapsing-remitting multiple sclerosis; HCs, healthy controls.

**Figure 4:**
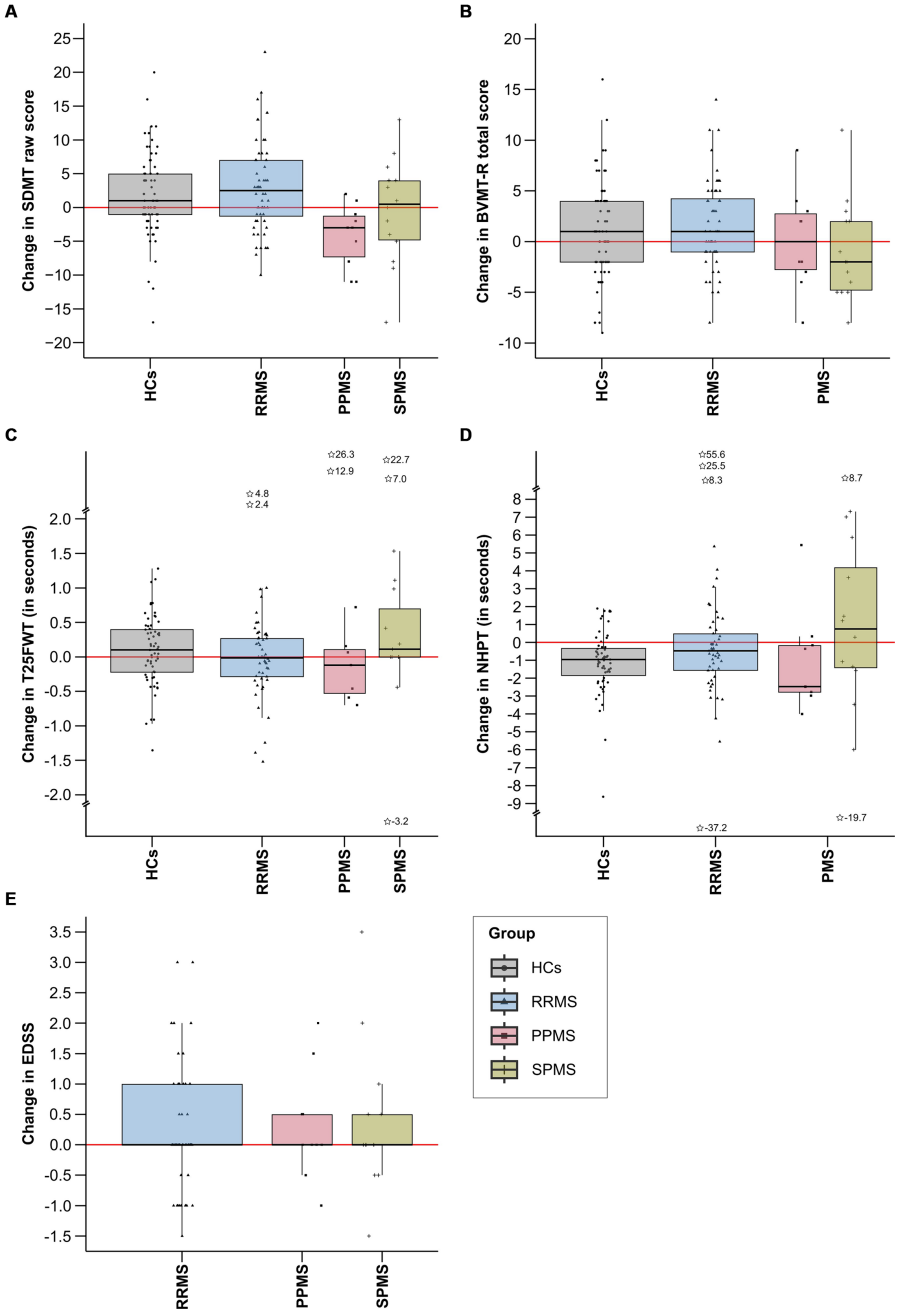
This figure displays the absolute change in each functional measure from baseline to latest follow-up by group. Total score changes for SDMT **(A)**, BVMT-R **(B)**, and EDSS **(E)** are included, while changes in completion time in seconds are provided for T25FWT **(C)** and NHPT **(D)**. Extreme values, defined as scores falling below the first quartile minus three times the interquartile range (1st quartile −3*IQR) or exceeding the third quartile plus three times the interquartile range (3rd quartile +3*IQR), are denoted by asterisks, alongside their precise numerical values. IQR, interquartile range; SDMT, symbol digit modalities test; BVMT-R, brief visuospatial memory test-revised; NHPT, nine-hole peg test; T25FWT, timed 25-foot walk test; EDSS, expanded disability status scale; PPMS, patients with primary progressive multiple sclerosis; SPMS, patients with secondary progressive multiple sclerosis; RRMS, patients with relapsing-remitting multiple sclerosis; HCs, healthy controls.

Table 1 shows the descriptive and clinical characteristics of all patients and HCs at baseline and latest follow-up. Performance on the SDMT, BVMT-R, NHPT, T25FWT, and EDSS at baseline and each follow-up are presented in Figure 3, illustrating substantial fluctuations across time in both patients as well as HCs. Figure 4 illustrates the individual absolute change in each functional measure from baseline to latest follow-up as well as a summary on the group level. While the median absolute change differed significantly from zero for some outcomes and groups (SDMT: all groups except SPMS; NHPT: HCs; BVMT-R: RRMS), on the group level, none of these changes surpassed the defined thresholds for clinically meaningful change in any outcome. Comparing the absolute change per outcome between groups (HCs, RRMS, PPMS, SPMS) revealed a significant difference in the absolute SDMT change between patients with PPMS and both RRMS (Bonferroni-corrected *p* = 0.009) and HCs (Bonferroni-corrected *p* = 0.03). None of the other outcomes differed significantly between groups.

**Table 1:** Sample characteristics at baseline and latest follow-up.

Characteristic	RRMS		SPMS		PPMS		HCs	
	BL (*n* = 56)	FU (*n* = 52)	BL (*n* = 14)	FU (*n* = 18)	BL (*n* = 10)	FU (*n* = 10)	BL (*n* = 69)	FU (*n* = 69)
Completed follow-ups, Md (IQR)^a^	2 (2)		2 (1)		1 (1)		2 (2)	
Time since baseline, Md (IQR), months	29 (20)		31 (17)		19 (18)		26 (22)	
Sex, *N* (%), female^b^	37 (66)	35 (67)	9 (64)	11 (61)	5 (50)	5 (50)	43 (62)	
Age, Md (IQR), years	39 (17)	40 (16)	49 (15)	52 (13)	57 (6)	58 (5)	36 (31)	37 (30)
Education, Md (IQR), years	15 (4)	16 (4)	15 (5)	15 (5)	16 (5)	16 (5)	16 (3)	17 (4)
Occupation, *N* (%), yes	43 (77)	43 (83)	5 (36)	7 (39)	6 (60)	4 (40)	61 (88)	58 (84)
Relapse activity throughout study, *N* (%), yes		13 (25)		6 (33)				
MEP latency, Md (IQR), ms^c^	22.69 (4)		24.64 (7)		26.21 (10)		22.56 (2)	
ΔPost-pre MEP amplitude, Md (IQR), mV	0.48 (0.51)		0.26 (0.54)		0.11 (0.82)		0.47 (0.58)	
**HADS, *N* (%), clinical** ^d^								
Anxiety	9 (16)	7 (14)	1 (7)	2 (11)	0 (0)	0 (0)	1 (1)	0
Depression	6 (11)	4 (8)	2 (14)	4 (22)	0 (0)	0 (0)	0	0
**FSMC, *N* (%), moderate or severe** ^e^								
Motor	26 (46)	26 (50)	14 (100)	17 (94)	6 (60)	8 (80)	4 (6)	6 (9)
Cognitive	23 (41)	22 (42)	11 (79)	13 (72)	3 (30)	4 (40)	7 (10)	5 (7)
Disease duration, Md (IQR), years	9 (12)	13 (12)	20 (23)	20 (17)	4 (7)	6 (6)		
EDSS, Md (IQR)^f^	1.5 (3)	2 (3)	5.5 (3)	6 (3)	4.75 (3)	4.25 (3)		
**DMT at time of assessment, *N* (%)** ^g^								
None	9 (16)	5 (10)	3 (21)	3 (17)	1 (10)	1 (10)		
Group 1	3 (5)	4 (8)	0	0 (0)	0 (0)	0 (0)		
Group 2	4 (7)	6 (12)	2	5 (28)	0 (0)	0 (0)		
Group 3	40 (71)	37 (71)	9	10 (56)	9 (90)	9 (90)		

Median and interquartile range are displayed for metric variables due to non-normal distribution in at least one group at one time of assessment. *n* = 4 RRMS patients converted to secondary progressive multiple sclerosis at latest follow-up. HCs, healthy controls; Md, median; IQR, interquartile range; MEP, motor evoked potential; HADS, hospital anxiety and depression scale; FSMC, fatigue scale of motor and cognition; EDSS, expanded disability status scale; DMT, disease-modifying therapy; BL, baseline; FU, follow-up.

a Remote assessment at latest follow-up: RRMS: *n* = 2, HCs: *n* = 2.

b Sex defined as sex assigned at birth.

c Missing data: RRMS: *n* = 4, PPMS: *n* = 1, SPMS: *n* = 2, HCs: *n* = 19.

d Defined as scores ≥11 (28). Missing data: HCs BL: *n* = 3.

e Defined based on cut-offs provided in the FSMC manual (29). Missing data: HCs BL: *n* = 3.

f Missing data: SPMS *n* = 1 at baseline.

g Groups based on the current guidelines in Germany (S2k-Leitlinie) (39): group 1 = beta-interferone, dimethyl fumarate, teriflunomide, glatirameroide, group 2 = cladribine, s1p-receptor modulators, group 3 = alemtuzumab, CD20-antibodies, natalizumab. One patient (SPMS) was on intravenous immunoglobulin therapy at BL and FU, which is currently not approved as a DMT in patients with MS in Germany. However, this patient presented with contraindications for immunotherapy and gammaglobuline deficiency. This treatment was assigned to group 2.

Analyzing individual data, *n* = 35 (69%) patients with MS compared to *n* = 16 (31%) HCs experienced decline in any of the functional outcomes (*p* = 0.008, Φ = 0.22). Table 2 presents the number of participants per group (HCs vs. MS) with stable, improved, and decline performance in each functional outcome.

**Table 2:** Number of participants with improved, stable, and declined performance in each functional outcome.

Parameter	MS (*n* = 80)			HCs (*n* = 69)		
	Improved	Stable	Declined	Improved	Stable	Declined
SDMT, *n* (%)	15 (19)	58 (73)	7 (9)	13 (19)	52 (75)	4 (6)
BVMT-R, *n* (%)	7 (9)	70 (88)	3 (4)	10 (15)	54 (78)	5 (7)
NHPT, *n* (%)^a^	4 (5)	63 (85)	7 (10)	1 (2)	61 (98)	0 (0)
T25FWT, *n* (%)^a^	3 (4)	62 (84)	9 (12)	2 (3)	53 (86)	7 (11)
EDSS, *n* (%)^b^	15 (19)	45 (57)	19 (24)			

MS, multiple sclerosis; HCs, healthy controls; SDMT, symbol digit modalities test; BVMT-R, brief visuospatial memory test-revised; NHPT, nine-hole peg test; T25FWT, timed 25-foot walk test; EDSS, expanded disability status scale.

a Missing data: *n* = 13 (7 HCs, 6 MS).

b Missing data due to missing EDSS at baseline: *n* = 1.

The EDSS exhibited the highest incidence of clinically relevant decline among the outcome measures, with *n* = 19 (24%) patients experiencing such decline. However, a comparable number of patients also demonstrated clinically relevant EDSS improvement (*n* = 15, 19%). In the T25FWT, *n* = 9 (12%) patients presented with clinically meaningful decline compared to *n* = 3 (4%) patients with clinically relevant improvement. Both the NHPT and the SDMT showed *n* = 7 (9%) patients with clinically meaningful decline, but more patients improved in the SDMT (*n* = 15, 19%) than in the NHPT (*n* = 4, 5%). Only *n* = 3 (4%) exceeded the cognitive decline cut-off on the BVMT-R, whereas *n* = 7 (9%) demonstrated clinical improvement.

Examining HCs, *n* = 7 (11%) experienced decline on the T25FWT, compared to *n* = 4 (6%), *n* = 5 (7%), and *n* = 0 on the SDMT, BVMT-R, and NHPT, respectively. Clinically relevant improvement was observed in *n* = 13 (19%) on the SDMT, *n* = 10 (15%) on the BVMT-R, *n* = 1 (2%) on the NHPT, and *n* = 2 (3%) on the T25FWT. Comparing the number of events of clinically relevant decline between HCs and patients with MS, significantly more patients than HCs experienced clinically relevant decline in the NHPT (*p* = 0.016, Φ = 0.21). No significant differences in the number of events were detected for all other outcomes.

### 3.2 Predictive value of QPS-induced plasticity for functional decline

**Figure 5:**
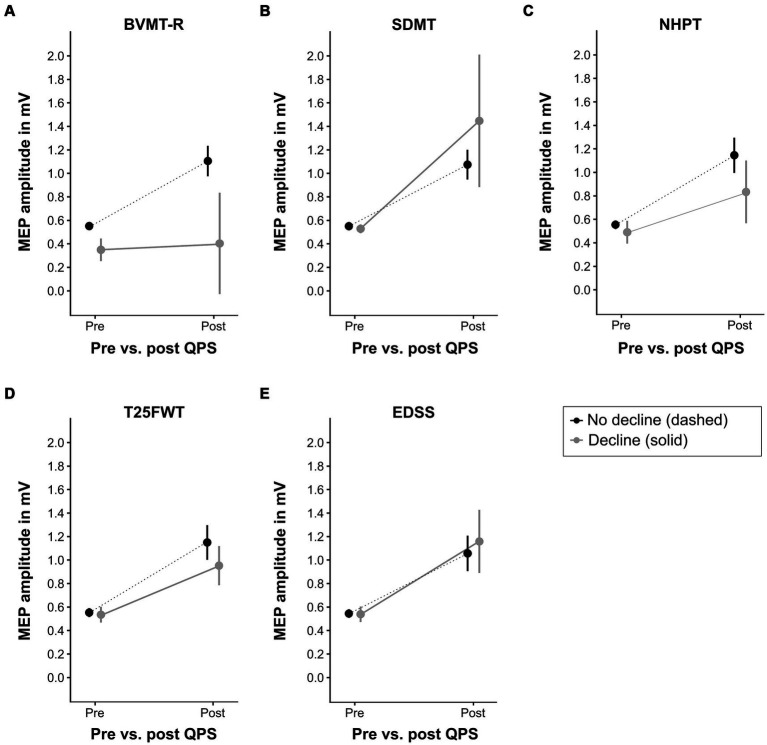
QPS-induced plasticity at baseline in patients with different clinical outcomes at latest follow-up. This figure displays the level of QPS-induced plasticity at baseline in patients with clinically relevant decline at latest follow-up (solid gray line) compared to patients without clinically relevant decline (dashed black line) in each functional outcome (**A** = BVMT-R, **B** = SDMT, **C** = NHPT, **D** = T25FWT, **E** = EDSS). Please refer to Table 2 for the number of patients per group. QPS, quadripulse stimulation; BVMT-R, brief visuospatial memory test-revised; SDMT, symbol digit modalities test; NHPT, nine-hole peg test; T25FWT, timed 25-foot walk test; EDSS, expanded disability status scale. MEP, motor evoked potential, mV, millivolts.

Figure 5 illustrates the increase of MEP amplitude following QPS in patients with clinically relevant decline in the functional outcome measures compared to those without clinically relevant decline. Increase in MEP amplitude following QPS was significantly lower in patients with clinically meaningful decline in the NHPT (*β* = −0.25, *p* = 0.02) and BVMT-R (*β* = −0.50, *p* = 0.005) compared to clinically stable or improved patients, as indicated by a significant QPS*group interaction. No significant differences were detected for progression in the SDMT (*β* = +0.39, *p* = 0.09), T25FWT (*β* = −0.18, *p* = 0.07), and EDSS (*β* = +0.11, *p* = 0.23). The final LMEMs for each functional outcome are presented in Supplementary Tables S1–S5.

Receiver-operating characteristic analysis revealed high accuracy of ΔMEP at baseline to differentiate between patients with and without clinically relevant decline in BVMT-R at latest follow-up (area under the curve = 0.853). For all other outcomes, ΔMEP at baseline could not discriminate between patients with vs. without clinically relevant decline.

Cox-proportional hazard models and logistic regression did not reveal associations between the degree of baseline plasticity and clinically relevant decline in any functional measure. Kaplan–Meier curves with the results of the Cox-Proportional Hazard models are presented in Supplementary Figure S1. Supplementary Figure S2 illustrates the receiver operating characteristic curves and the odds ratios (including 95% confidence intervals) of clinically meaningful decline are presented in Supplementary Table S6.

## 4 Discussion

To the best of our knowledge, this is the first study investigating the prognostic value of QPS-induced plasticity in patients with MS. In this cohort of MS patients, only a small number of patients exhibited clinically meaningful declines in the SDMT, BVMT-R, NHPT, T25FWT, and EDSS during a median observational period of 2 years. Importantly, the number of patients demonstrating clinically meaningful change did not significantly differ from HCs, except for the NHPT. However, comparing patients with clinically relevant functional decline in the BVMT-R and NHPT using LMEM revealed significantly lower levels of baseline plasticity in patients with functional decline in these measures. Receiver operating characteristic analysis indicated predictive utility of baseline synaptic plasticity in discerning between patients experiencing functional decline and those maintaining stability or presenting improvement only for the BVMT-R. Other statistical approaches, such as Cox proportional Hazard models and logistic regression, did not reveal significant differences between groups and no association between baseline plasticity and functional decline was observed for the T25FWT, SDMT and EDSS.

Our results confirm previous studies showing that both cognitive and physical disability progression occur slowly in patients with MS and assessment of cognitive function appears to be volatile. A previous study examining a 5 years follow-up period reported cognitive decline in 28% of MS patients, with a higher incidence observed among PMS compared to RRMS patients (40). Even after 10 years of follow-up, clinically relevant changes in cognitive or physical disability at the group level have been reported to be scarce, with only 24% of patients displaying cognitive decline (41). Another study also spanning a 10 years follow-up period reported a 10% increase in the overall proportion of MS patients exhibiting cognitive impairment. Notably, the authors reported a dynamic pattern of cognitive function, where some patients demonstrated cognitive improvement in specific domains, while others experienced impairment in different cognitive domains during follow-up compared to baseline assessments (42). However, contrasting reports of higher rates of cognitive decline—50% and 62% over periods of 6 and 7 years, respectively—have also been documented (43, 44). Consequently, the literature presents considerable variations in rates of cognitive decline over time, influenced by factors such as patients’ demographics, definitions of cognitive decline, applied neuropsychological test batteries, frequency of assessments, and duration of follow-up. Another explanation for the low rate of clinically relevant deterioration in our cohort may be the high percentage of patients on high efficacy therapy. This is a common phenomenon in cohorts of patients recruited at tertiary referral centers.

Importantly, we report a higher proportion of patients exhibiting improvement in cognitive tests compared to those demonstrating decline. This aligns with recent data from a study involving RRMS patients treated with subcutaneous daclizumab or intramuscular interferon beta-1a, reporting more frequent improvement in the paced auditory serial addition test and SDMT than decline 144 weeks post-baseline assessment. This phenomenon might be attributed to practice effects in the previous study as participants were tested every 6 months (45). However, we also did not observe a significant difference in decline between MS patients and HCs in the SDMT or the BVMT-R despite less frequent testing and the use of alternative forms for the BVMT-R at each assessment. Conversely, clinically relevant decline was more prevalent than improvement in motor function outcomes for patients with MS, and significantly more patients with MS experienced decline in the NHPT than HCs. These findings underscore the necessity for further research on reliable change indexes in neuropsychological tests, especially in the context of annual assessments.

Notable fluctuations have also been described for repeated assessment using the EDSS. In a randomized controlled trial, 21% of patients receiving a placebo exhibited significant improvement on the EDSS during a 5 years follow-up, while 25% experienced significant worsening. Conversely, patients receiving immunotherapy with cladribine demonstrated improvement in 18% and worsening in 16% of cases (46). In our cohort, we observed a similar trend, with 24% of patients experiencing a clinically relevant decline in EDSS scores compared to 19% showing improvement. These findings align with existing literature and highlight concerns regarding the reliability and sensitivity of the EDSS to detect meaningful changes over time (30). Notably, one criticism of the EDSS is its emphasis on ambulation issues for scores ≥6, often overlooking other important functional deficits (47).

The LMEM revealed significant differences in baseline plasticity between patients with and without meaningful decline only for the BVMT-R and NHPT, but not for the SDMT, T25FWT, and the EDSS. Regarding the SDMT, this observation may stem from its lack of specificity, as noted in previous research (48). Despite its sensitivity to detect cognitive impairment in patients with MS, the SDMT lacks specificity, since a patient’s performance on this test does not only rely on cognitive processing speed but also involves other cognitive aspects such as working memory, paired-associate learning, and visual scanning, albeit to a lesser extent (49). In contrast, the BVMT-R serves as a sensitive measure of learning and memory, less prone to confounding other cognitive functions. However, it may be marginally influenced by manual impairments owing to the drawing component of the test (48). This may explain the congruence in LMEM results between the NHPT and BVMT-R. Two out of three patients exhibiting decline on the BVMT-R also demonstrated decline on the NHPT, which assessed manual dexterity of the dominant hand in all cases. Nonetheless, the differential results for the SDMT and BVMT-R contrast with our previous cross-sectional results, which revealed significant correlations between QPS-induced plasticity and performance on both the BVMT-R and SDMT in patients with RRMS and those with normal cortical latency (16, 17).

One possible explanation could be the consistent use of the same form of the SDMT throughout the study due to the lack of normative data for alternate forms in German-speaking populations. In contrast, alternate forms were used for the BVMT-R. While the cross-sectional correlations were based on a single assessment of the SDMT and BVMT-R, the current study evaluated changes over time in these tests. The repeated administration of the same SDMT form may have influenced these changes as using identical forms has been shown to predict improved performance, especially when the time intervals between tests are less than 2 years. Consequently, it has been proposed that maintaining an unchanged SDMT score up to the fifth annual assessment using the same form suggests impairment (50). Considering the novelty and uncertain clinical significance of this finding, we opted to adhere to established thresholds for clinically meaningful change. However, the criterion of ≥8 points change on the SDMT has been established in a study with longer re-test intervals and less frequent neuropsychological evaluations compared to our investigation (32). Therefore, we may have missed clinically relevant cognitive decline on the SDMT. Importantly, the limited number of patients showing decline on the BVMT-R warrants consideration as well.

Regarding changes in the T25FWT, recent research affirmed the clinical utility of a 20% change cutoff (45). The observation that a higher proportion of patients experienced clinically meaningful decline compared to improvement (12% vs. 4%) in our cohort further supports the validity of this cutoff. The absence of an association between baseline plasticity and clinical decline on the T25FWT aligns with our earlier discovery of no cross-sectional correlation with QPS-induced plasticity (16). As discussed previously, this may be explained by the test’s susceptibility to influences from spinal and cerebellar lesions, aspects potentially not fully captured by QPS. Furthermore, the T25FWT primarily measures walking speed, whereas tests such as the two-minute walk test and 6 min walk test (6MWT) assess walking distance (51). Although there is a general correlation between these shorter and longer walking tests, higher gait velocities have been reported on short tests compared to long tests (52), and the T25FWT has been discussed in the context of floor effects (53). Additionally, estimation errors when predicting 6MWT performance based on the T25FWT were higher in patients with MS and moderate disability compared to those with mild disability (52). Therefore, subtle motor changes may have not been detected in mildly affected patients in our study.

Importantly, this study exclusively examined QPS-5-induced plasticity, evaluating LTP-like synaptic plasticity, which is a rapid-onset mechanism of neuroplasticity (15, 54), However, focusing solely on these rapid-onset mechanisms might not comprehensively capture all facets of neuroplasticity. Other parts of plasticity, e.g., LTD-like plasticity may also be relevant, considering the potential involvement of inhibitory circuits in MS (55). Rather than isolating either of these two synaptic plasticity types, their interplay could be crucial. In fact, our previous study indicated that stabilizing metaplasticity during relapses might be more pivotal than LTP-like plasticity itself in preventing motor disability (16).

Furthermore, we did not account for brain resilience, which refers to any concept associated with the brain’s ability to sustain cognition and function amidst aging and disease (56). Among these concepts, cognitive reserve proposes that the brain actively copes with damage by leveraging pre-existing cognitive processes or by engaging compensatory mechanisms (57). Another pertinent concept is brain reserve, primarily referring to passive neurobiological measures like brain volume and synaptic count (58). Consequently, high cognitive and/or brain reserve enable (cognitive) performance to exceed expectations considering the degree of brain changes and/or injury (56). Therefore, low levels of synaptic plasticity might not correlate with functional decline in certain patients due to elevated levels of cognitive and/or brain reserve. While we did not assess brain reserve, various proxies for cognitive reserve exist, with education level being one of them (56). In our study, the median years of education at baseline were rather high in the overall patient group (15 years), indicating high levels of cognitive reserve, which could explain the observed low prevalence of cognitive decline. However, cognitive rehabilitation, potentially taking place between follow-ups, may have influenced our outcome measures as well. Emerging evidence indicates that patients with MS, particularly those with RRMS, the predominant subgroup in our cohort, may benefit from such interventions (59).

Initially, the annual follow-up plan included not only clinical assessments but also repeated assessments of QPS-5 to analyze changes in the degree of plasticity. However, due to unforeseen circumstances such as the COVID-19 pandemic and technical defects of the TMS coils and signal amplifier, the application of QPS-5 had to be discontinued. Consequently, we could not analyze longitudinal changes in plasticity. However, it is possible that plasticity is influenced by disease activity, brain resilience, and their interaction. Given this presumably dynamic nature of plasticity, changes in plasticity may have occurred between baseline and follow-up, possibly in both directions (enhanced and diminished plasticity).

Expanding on our earlier finding underscoring the importance of evaluating synaptic plasticity within the context of corticospinal tract integrity among MS patients (16), our goal was to conduct subgroup analyses to assess the predictive value of QPS-induced plasticity in patients with pathological latency compared to those with normal latency. Due to the low number of events, i.e., patients experiencing functional decline, this was not feasible. Additionally, given the relatively small number of patients with PMS, and especially PPMS, we decided against performing subtype specific analyses.

Despite these limitations, we present a large cohort of MS patients with longitudinal clinical follow-ups and baseline plasticity assessment using QPS-5. Moreover, the inclusion of closely matched HCs allows for a comparison of changes observed between MS patients and HCs. We conclude that LTP-like synaptic plasticity may be of functional relevance in patients with MS and that more research is needed to identify and better define reliable change in cognitive performance in these patients.

## Data availability statement

The raw data supporting the conclusions of this article will be made available by the authors, without undue reservation.

## Ethics statement

The studies involving humans were approved by Ethical Committee of the Medical Faculty of the Heinrich Heine University Düsseldorf. The studies were conducted in accordance with the local legislation and institutional requirements. The participants provided their written informed consent to participate in this study.

## Author contributions

CB: Writing – review & editing, Writing – original draft, Visualization, Project administration, Methodology, Investigation, Formal analysis, Data curation, Conceptualization. LJ: Writing – review & editing, Investigation, Formal analysis. CH: Writing – review & editing, Investigation. SM: Writing – review & editing, Resources. AS: Writing – review & editing, Resources. I-KP: Writing – review & editing, Writing – original draft, Methodology, Funding acquisition, Conceptualization. PA: Writing – review & editing, Writing – original draft, Methodology, Investigation, Funding acquisition, Conceptualization.
