# Supplementary material for: Predictive value of synaptic plasticity for functional decline in patients with multiple sclerosis
Source: Front Neurol. 2024 Jun 21;15:1410673. doi: 10.3389/fneur.2024.1410673 (PMC11224454; doi:10.3389/fneur.2024.1410673)
Supplement: Supplementary file 1 [file Data_Sheet_1.docx]

Supplementary Material

# Supplementary Methods. Remote assessment

Baseline assessments were conducted in-person, while video-based neuro(psycho)logical testing was offered for follow-up evaluations. This option was provided only as a last resort for participants unable or unwilling to attend in-person assessments. Prior to remote assessments, participants received an email containing an uniform resource locator for an online video meeting. Adequate audio and video quality were required, with participants asked to minimize potential distractions, such as interruptions from family members, or phone calls.

At the beginning of each remote assessment, trained personnel conducted a structured interview to assess medical history and approximate the Expanded Disability Status Scale (EDSS) score. Patient-reported EDSS assessments and those determined by neurologists highly correlate, suggesting patient-reported evaluations as valid alternatives when physician-derived scores are unavailable (1). Modifications to one of these questionnaires (2) were made to simplify administration, including incorporation into a structured interview format and detailed assessment of functional systems.

Following the interview, cognitive tests were administered. Previous studies have shown remote administration of the Symbol Digit Modalities Test (SDMT) to yield comparable results to in-person testing, recommending it as a valid option for virtual assessment of information-processing speed (3–5).

Regarding the Brief Visuospatial Memory Test-Revised (BVMT-R), only one study has examined its virtual administration, reporting significantly higher scores in the remote setting compared to in-person testing (5). Possible factors contributing to this disparity include the use of different investigators and varied screen sizes among participants (phones vs. computer screens). In our study, investigators remained consistent across administration settings, and participants were required to have a minimum screen size of either a tablet or computer for participation.

Cognitive tests were presented via screen-sharing as PDF documents. SDMT instructions mirrored those of in-person assessments. For the BVMT-R, participants were instructed to have necessary materials prepared, including three blank sheets of DIN A4 paper, a dark pen, and an eraser. Following each trial, a screenshot of the response sheet displayed to the webcam was captured to prevent any subsequent alterations. After each trial, participants were reminded to keep their previous responses out of sight. Scoring of the geometric shapes was conducted post-assessment, adhering to the same criteria employed in in-person evaluations.

# Supplementary Table 1. Multivariable linear mixed-effects model of MEP amplitude pre and post QPS at baseline in patients with clinically significant decline in the BVMT-R

| **Fixed Effects** | | | | | **Random Effects** | |
| --- | --- | --- | --- | --- | --- | --- |
|  | *β-coefficient (95% CI)* | *SE_b_* | *t-value* | *p* | | *SD* |
| Intercept | +0.55 (+0.51; +0.58)^a^ | 0.02 | +34.00 | **<.0001** | |  |
| Pre QPS | Reference |  |  |  | |  |
| Post QPS | +0.55 (+0.43; +0.68)^a^ | 0.07 | +8.80 | **<.0001** | |  |
| Stable or improved BVMT-R | Reference |  |  |  | |  |
| Declined BVMT-R | -0.20 (-0.31; -0.10)^a^ | 0.05 | -3.84 | **.0003** | |  |
| Fatigue | -0.05 (-0.08; -0.03)^a^ | 0.01 | -4.41 | **<.0001** | |  |
| Post QPS*Declined BVMT-R | -0.50 (-∞; -0.19)^a^ | 0.19 | -2.67 | **.005** | |  |
| Subject*Pre QPS |  |  |  |  | | 0.09 |
| Subject*Post QPS |  |  |  |  | | 0.38 |
| Residual |  |  |  |  | | 0.11 |

*Note.* Two-tailed 95% confidence intervals and *p*-values are displayed for all factors except for the primary variable of interest (Post QPS*Declined BVMT-R). For this factor, testing our hypothesis that patients with functional decline present with lower levels of QPS-induced plasticity at baseline than patients with stable performance, one-tailed 95% confidence intervals and *p*-values are reported. *p*-values <.05 are in boldface. *t*- and *p*-values are based on asymptotic Wald test. Fatigue centered at sample mean. R²(conditional)=0.93. R²(marginal)=0.50. Adjusted Intraclass Correlation Coefficient=0.86.

MEP= Motor evoked potential. QPS= Quadripulse stimulation. BVMT-R= Brief Visuospatial Memory Test – Revised.

^a^ indicates statistical significance.

# Supplementary Table 2. Multivariable linear mixed-effects model of MEP amplitude pre and post QPS at baseline in patients with clinically significant decline in the NHPT

| **Fixed Effects** | | | | | **Random Effects** | |
| --- | --- | --- | --- | --- | --- | --- |
|  | *β-coefficient (95% CI)* | *SE_b_* | *t-value* | *p* | | *SD* |
| Intercept | +0.55 (+0.52; +0.59)^a^ | 0.02 | +29.41 | **<.0001** | |  |
| Pre QPS | Reference |  |  |  | |  |
| Post QPS | +0.59 (+0.45; +0.73)^a^ | 0.07 | +8.37 | **<.0001** | |  |
| Stable or improved NHPT | Reference |  |  |  | |  |
| Declined NHPT | -0.06 (-0.17; +0.04) | 0.05 | -1.22 | .23 | |  |
| Post QPS*Declined NHPT | -0.25 (-∞; -0.05) | 0.12 | -2.06 | **.02** | |  |
| Subject*Pre QPS |  |  |  |  | | 0.12 |
| Subject*Post QPS |  |  |  |  | | 0.35 |
| Residual |  |  |  |  | | 0.11 |

*Note.* Two-tailed 95% confidence intervals and *p*-values are displayed for all factors except for the primary variable of interest (Post QPS*Declined NHPT). For this factor, testing our hypothesis that patients with functional decline present with lower levels of QPS-induced plasticity at baseline than patients with stable performance, one-tailed 95% confidence intervals and *p*-values are reported. *p*-values <.05 are in boldface. *t*- and *p*-values are based on asymptotic Wald test. R²(conditional)=0.99. R²(marginal)=0.57. Adjusted Intraclass Correlation Coefficient=0.98.

MEP= Motor evoked potential. QPS= Quadripulse stimulation. NHPT= Nine-hole peg test.

^a^ indicates statistical significance.

# Supplementary Table 3. Multivariable linear mixed-effects model of MEP amplitude pre and post QPS at baseline in patients with clinically significant decline in the SDMT

| **Fixed Effects** | | | | | **Random Effects** | |
| --- | --- | --- | --- | --- | --- | --- |
|  | *β-coefficient (95% CI)* | *SE_b_* | *t-value* | *p* | | *SD* |
| Intercept | +0.55 (+0.51; +0.59)^a^ | 0.02 | +29.50 | **<.0001** | |  |
| Pre QPS | Reference |  |  |  | |  |
| Post QPS | +0.52 (+0.41; +0.64)^a^ | 0.06 | +8.69 | **<.0001** | |  |
| Stable or improved SDMT | Reference |  |  |  | |  |
| Declined SDMT | -0.02 (-0.07; +0.03) | 0.03 | -0.80 | .43 | |  |
| Latency | -0.05 (-0.08; -0.02) ^a^ | 0.02 | -3.01 | **.004** | |  |
| Post QPS*Declined SDMT | +0.39 (-∞; +0.87) | 0.29 | +1.36 | .09 | |  |
| Post QPS*Latency | -0.22 (-0.33; -0.11)^a^ | 0.06 | -3.76 | **.0004** | |  |
| Subject*Pre QPS |  |  |  |  | | 0.04 |
| Subject*Post QPS |  |  |  |  | | 0.47 |
| Residual |  |  |  |  | | 0.02 |

*Note.* Two-tailed 95% confidence intervals and *p*-values are displayed for all factors except for the primary variable of interest (Post QPS*Declined SDMT). For this factor, testing our hypothesis that patients with functional decline present with lower levels of QPS-induced plasticity at baseline than patients with stable performance, one-tailed 95% confidence intervals and *p*-values are reported. *p*-values <.05 are in boldface. *t*- and *p*-values are based on asymptotic Wald test. R²(conditional)=0.99. R²(marginal)=0.52. Adjusted Intraclass Correlation Coefficient=0.96. Latency centered at sample mean. Correlation structure: AR(1), phi=0.86.

MEP= Motor evoked potential. QPS= Quadripulse stimulation. SDMT= Symbol Digit Modalities Test.

^a^ indicates statistical significance.

# Supplementary Table 4. Multivariable linear mixed-effects model of MEP amplitude pre and post QPS at baseline in patients with clinically significant decline in the EDSS

| **Fixed Effects** | | | | | **Random Effects** | |
| --- | --- | --- | --- | --- | --- | --- |
|  | *β-coefficient (95% CI)* | *SE_b_* | *t-value* | *p* | | *SD* |
| Intercept | +0.54 (+0.51; +0.58)^a^ | 0.02 | +28.57 | **<.0001** | |  |
| Pre QPS | Reference |  |  |  | |  |
| Post QPS | +0.51 (+0.37; +0.65)^a^ | 0.07 | +7.14 | **<.0001** | |  |
| Stable or improved EDSS | Reference |  |  |  | |  |
| Declined EDSS | -0.01 (-0.08; +0.07) | 0.04 | -0.14 | .89 | |  |
| Fatigue | -0.04 (-0.07; -0.01)^a^ | 0.02 | -2.45 | **.02** | |  |
| Post QPS*Declined EDSS | +0.11 (-∞; +0.35) | 0.15 | +0.73 | .23 | |  |
| Subject*Pre QPS |  |  |  |  | | 0.11 |
| Subject*Post QPS |  |  |  |  | | 0.59 |
| Residual |  |  |  |  | | 0.10 |

*Note.* Two-tailed 95% confidence intervals and *p*-values are displayed for all factors except for the primary variable of interest (Post QPS*Declined EDSS). For this factor, testing our hypothesis that patients with functional decline present with lower levels of QPS-induced plasticity at baseline than patients with stable performance, one-tailed 95% confidence intervals and *p*-values are reported. *p*-values <.05 are in boldface. *t*- and *p*-values are based on asymptotic Wald test. R²(conditional)=0.96. R²(marginal)=0.29. Adjusted Intraclass Correlation Coefficient=0.95. Fatigue centered at sample mean and based on the total score on the Fatigue Scale for Motor and Cognitive Functions.(6)

MEP= Motor evoked potential. QPS= Quadripulse stimulation. EDSS= Expanded Disability Status Scale.

^a^ indicates statistical significance.

# Supplementary Table 5. Multivariable linear mixed-effects model of MEP amplitude pre and post QPS at baseline in patients with clinically significant decline in the T25FWT

| **Fixed Effects** | | | | | **Random Effects** | |
| --- | --- | --- | --- | --- | --- | --- |
|  | *β-coefficient (95% CI)* | *SE_b_* | *t-value* | *p* | | *SD* |
| Intercept | +0.55 (+0.51; +0.59)^a^ | 0.02 | +28.01 | **<.0001** | |  |
| Pre QPS | Reference |  |  |  | |  |
| Post QPS | +0.60 (+0.46; +0.74)^a^ | 0.07 | +8.39 | **<.0001** | |  |
| Stable or improved T25FWT | Reference |  |  |  | |  |
| Declined T25FWT | -0.02 (-0.10; +0.06) | 0.04 | -0.46 | .65 | |  |
| Latency | -0.06 (-0.09; -0.03)^a^ | 0.02 | -3.66 | **.0005** | |  |
| Post QPS*Decline T25FWT | -0.18 (-∞; +0.02) | 0.12 | -1.47 | .07 | |  |
| Post QPS* Latency | -0.23 (-0.33; -0.13)^a^ | 0.05 | -4.55 | **<.0001** | |  |
| Subject*Pre QPS |  |  |  |  | | 0.09 |
| Subject*Post QPS |  |  |  |  | | 0.22 |
| Residual |  |  |  |  | | 0.12 |

*Note.* Two-tailed 95% confidence intervals and *p*-values are displayed for all factors except for the primary variable of interest (Post QPS*Declined T25FWT). For this factor, testing our hypothesis that patients with functional decline present with lower levels of QPS-induced plasticity at baseline than patients with stable performance, one-tailed 95% confidence intervals and *p*-values are reported. *p*-values <.05 are in boldface. *t*- and *p*-values are based on asymptotic Wald test. R²(conditional)=0.92. R²(marginal)=0.78. Adjusted Intraclass Correlation Coefficient=0.63. Latency centered at sample mean. Correlation structure: AR(1), phi=0.55

MEP= Motor evoked potential. QPS= Quadripulse stimulation. T25FWT= Timed 25-foot walk test.

^a^ indicates statistical significance.

# Supplementary Table 6. Odds ratio (including 95% confidence intervals) of clinically meaningful decline for all functional outcomes.

|  | *Odds ratio* | *Lower bound CI* | *Upper bound CI* | *p* |
| --- | --- | --- | --- | --- |
| EDSS | 1.635 | 0.648 | 4.122 | .30 |
| SDMT | 1.671 | 0.531 | 5.263 | .38 |
| BVMT-R | 0.008 | 0.000 | 2.329 | .10 |
| NHPT | 0.185 | 0.018 | 1.938 | .16 |
| T25FWT | 0.134 | 0.015 | 1.236 | .08 |

*Note.* Two-tailed 95% confidence intervals and *p*-values are displayed.
EDSS= Expanded Disability Status Scale. SDMT= Symbol Digit Modalities Test. BVMT-R= Brief Visuospatial Memory Test – Revised. T25FWT = Timed 25-foot walk test. NHPT= Nine-hole peg test.

# Supplementary Figure 1

*
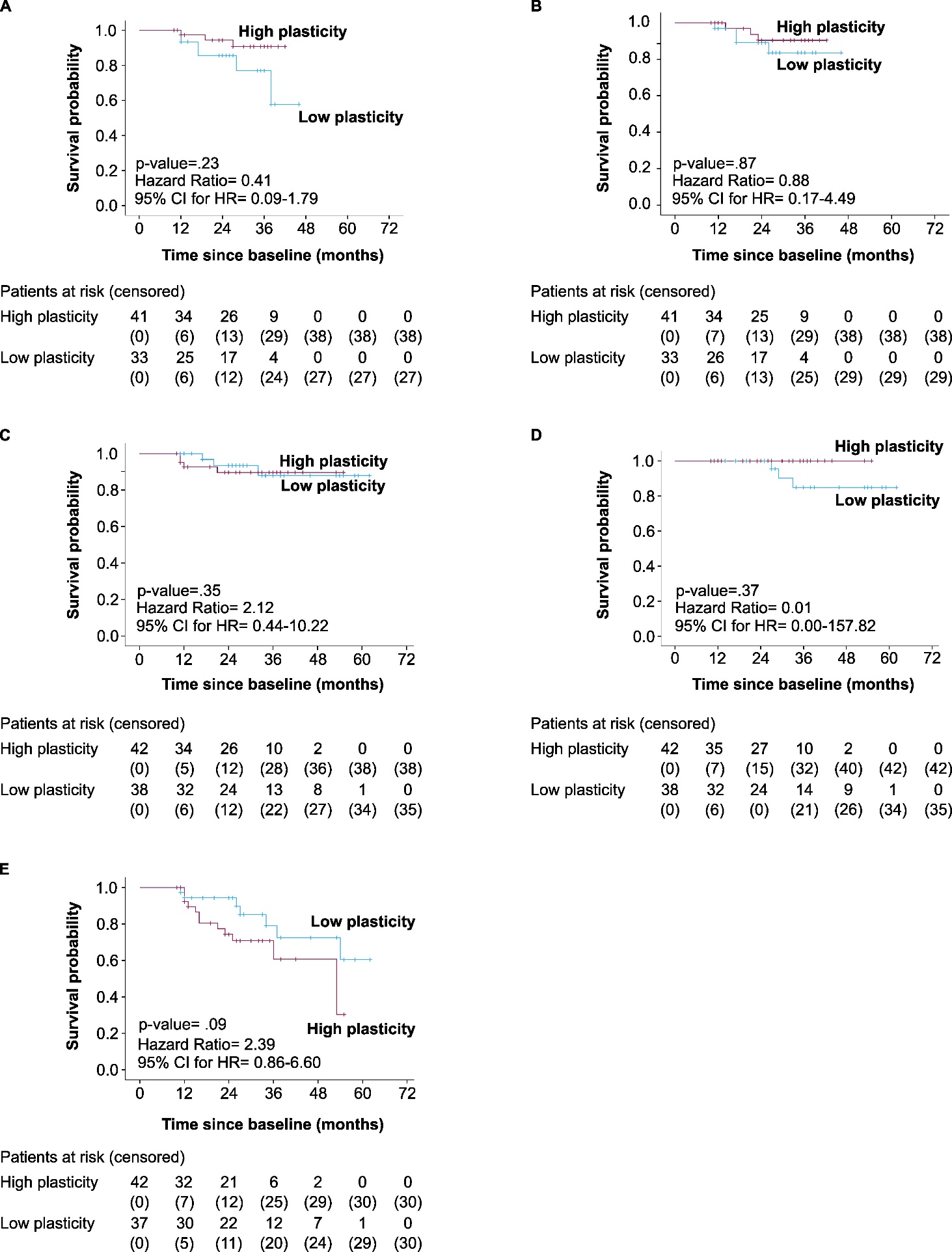
*

*Note*. This figure illustrates the Kaplan-Meier-Curves, Cox proportional hazard ratios, and number of remaining patients under observation for T25FWT (A), NHPT (B), SDMT (C), BVMT-R (D), and EDSS (E) from baseline to latest follow-up, stratified by high (red) vs. low (blue) plasticity at baseline. Survival probability (y-axis) represents the probability of not experiencing clinically relevant decline in the functional parameter. Age and sex were covariates in all models except for BVMT-R due to non-convergence when including these covariates.
T25FWT = Timed 25-foot walk test. NHPT= Nine-hole peg test. SDMT= Symbol Digit Modalities Test. BVMT-R= Brief Visuospatial Memory Test – Revised. EDSS= Expanded Disability Status Scale.

# Supplementary Figure 2


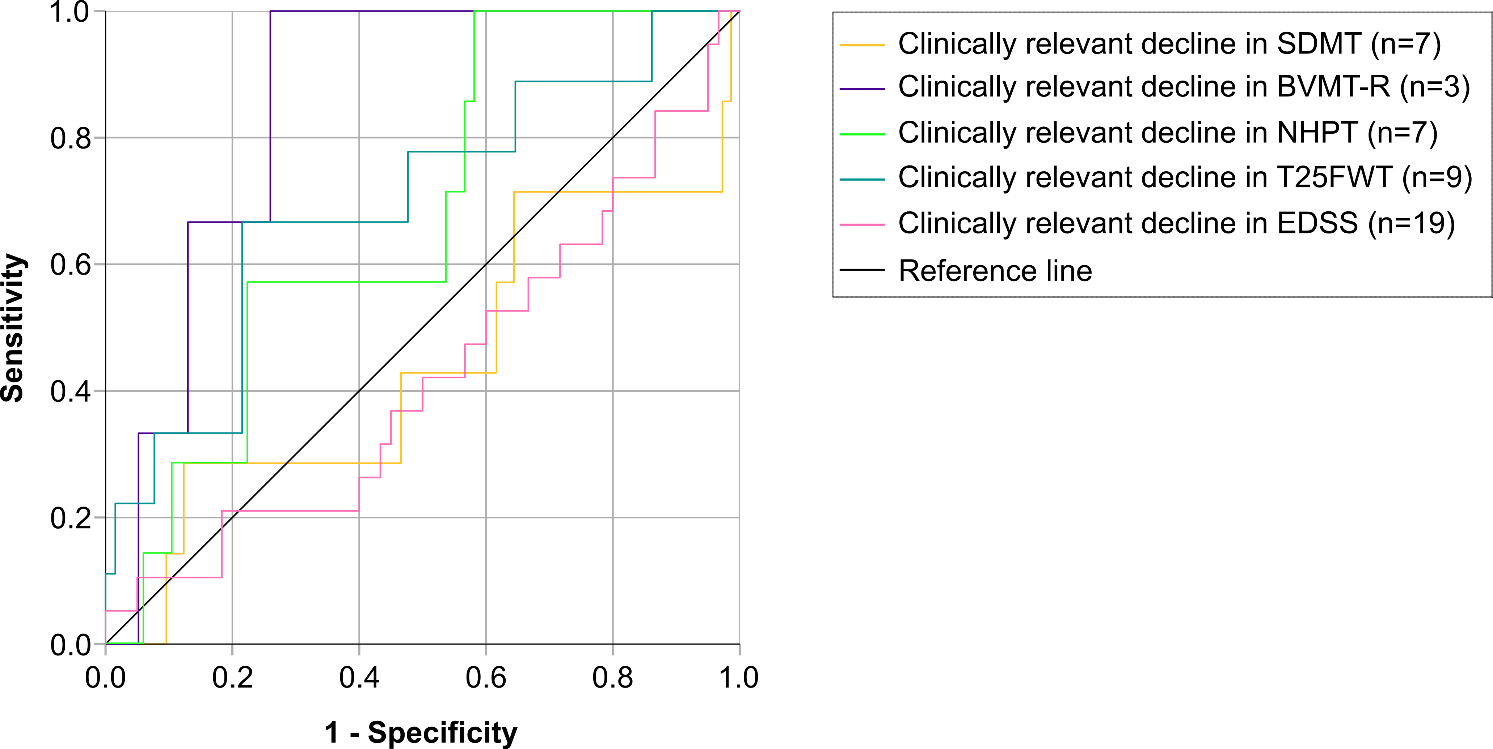


*Note*. This figure illustrates the receiver-operating characteristic curve of the accuracy of the difference between the maximum of the six mean post MEPs and the pre MEP amplitude to differentiate between patients with and without clinically relevant decline in the SDMT, BVMT-R, NHPT, T25FW, and EDSS. Area under the curve: SDMT=.442, BVMT-R=.853, NHPT= .672, T25FW= .697, EDSS= .425.
MEP= Motor evoked potential; SDMT= Symbol Digit Modalities Test; BVMT-R= Brief Visuospatial Memory Test – Revised; NHPT= Nine-hole peg test; T25FWT= Timed 25-foot walk test; EDSS= Expanded Disability Status Scale

# Supplementary Figure 3

*
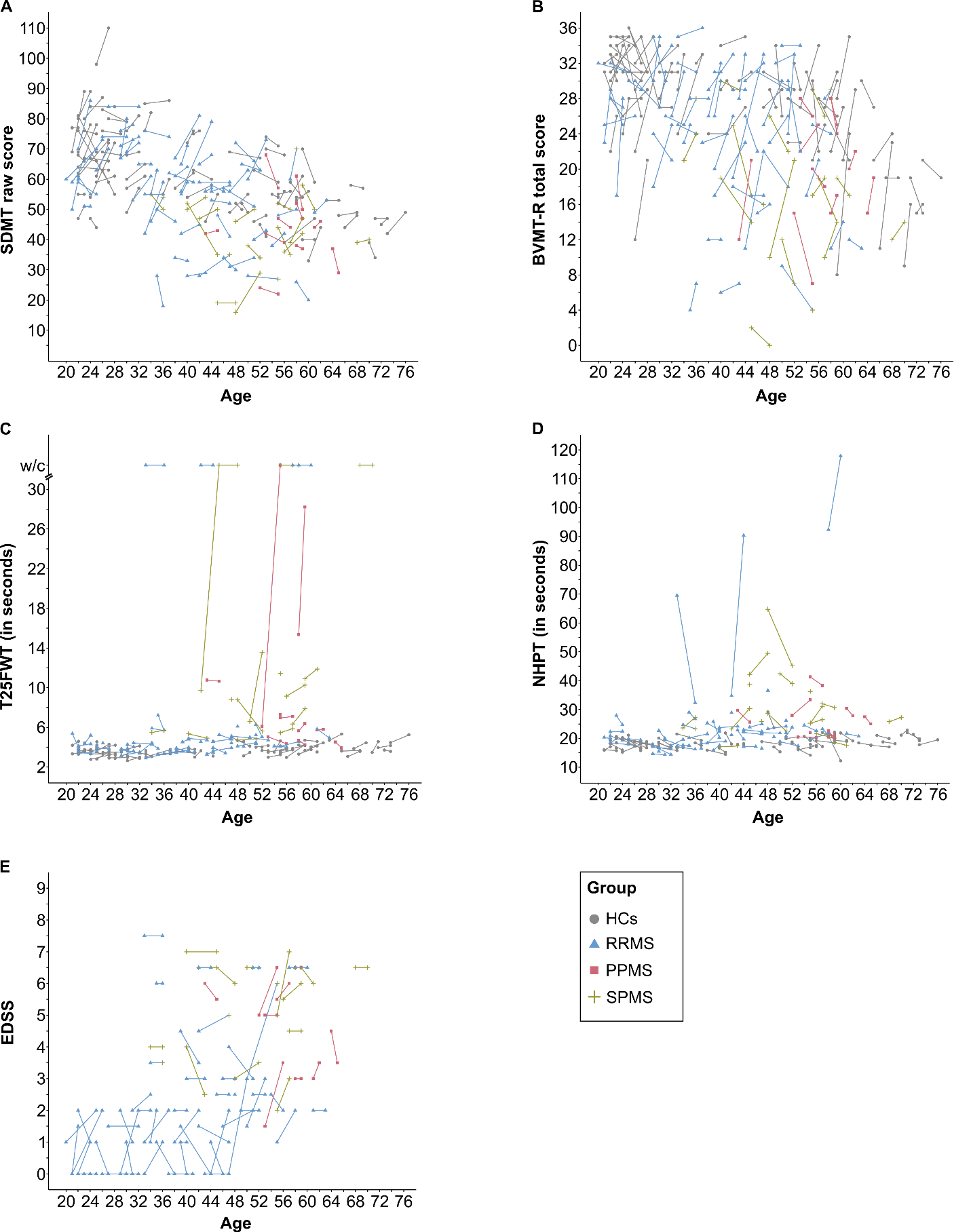
Note.* This figure displays the raw data for each assessment at baseline and last follow-up per subject for the SDMT (A), BVMT-R (B), NHPT (C), T25FWT (D), and EDSS (E). Lines connect data from the same subject. EDSS was missing for one patient at baseline.

SDMT= Symbol Digit Modalities Test; BVMT-R= Brief Visuospatial Memory Test – Revised; NHPT= Nine-hole peg test; T25FWT= Timed 25-foot walk test; w/c= wheelchair; EDSS= Expanded Disability Status Scale; PPMS= Patients with primary progressive multiple sclerosis; SPMS= Patients with secondary progressive multiple sclerosis; RRMS= Patients with relapsing-remitting multiple sclerosis; HCs= Healthy controls

Literature Cited

1. Collins C de, Ivry B, Bowen JD, Cheng EM, Dobson R, Goodin DS et al. A comparative analysis of Patient-Reported Expanded Disability Status Scale tools. Mult Scler 2016; 22(10):1349–58.

2. Goodin DS. A questionnaire to assess neurological impairment in multiple sclerosis. Mult Scler 1998; 4(5):444–51.

3. Barcellos LF, Horton M, Shao X, Bellesis KH, Chinn T, Waubant E et al. A validation study for remote testing of cognitive function in multiple sclerosis. Mult Scler 2021; 27(5):795–8.

4. Eilam-Stock T, Shaw MT, Sherman K, Krupp LB, Charvet LE. Remote administration of the symbol digit modalities test to individuals with multiple sclerosis is reliable: A short report. Mult Scler J Exp Transl Clin 2021; 7(1):2055217321994853.

5. Rogers F, Bane E, Dwyer CP, Alvarez-Iglesias A, Joyce RA, Hynes SM. Remote administration of BICAMS measures and the Trail-Making Test to assess cognitive impairment in multiple sclerosis. Neuropsychol Rehabil 2023; 33(5):903–26.

6. Penner IK, Raselli C, Stöcklin M, Opwis K, Kappos L, Calabrese P. The Fatigue Scale for Motor and Cognitive Functions (FSMC): validation of a new instrument to assess multiple sclerosis-related fatigue. Mult Scler 2009; 15(12):1509–17.
